# Large cystic lymphangioma of the pancreas: unusual finding with differential diagnosis and therapeutic considerations

**DOI:** 10.1093/jscr/rjad676

**Published:** 2023-12-19

**Authors:** Nikolaos Tasis, Eleni Mpalampou, Aikaterini Sarafi, Evangelia Skafida, Ioannis Tsouknidas, Evangelos Fradelos, Dimitrios K Manatakis, Dimitrios P Korkolis

**Affiliations:** Department of Surgical Oncology, General Anticancer and Oncological Hospital of Athens “Saint Savvas”, Athens, Greece; 2nd Department of Surgery, Athens Naval and Veterans Hospital, Athens, Greece; Department of Surgical Oncology, General Anticancer and Oncological Hospital of Athens “Saint Savvas”, Athens, Greece; Department of Surgical Oncology, General Anticancer and Oncological Hospital of Athens “Saint Savvas”, Athens, Greece; Department of Pathology, General Anticancer and Oncological Hospital of Athens “Saint Savvas”, Athens, Greece; Department of General Surgery, Lankenau Medical Center, Wynnewood, PA, United States; 2nd Department of Surgery, Athens Naval and Veterans Hospital, Athens, Greece; 2nd Department of Surgery, Athens Naval and Veterans Hospital, Athens, Greece; Department of Surgical Oncology, General Anticancer and Oncological Hospital of Athens “Saint Savvas”, Athens, Greece

**Keywords:** pancreatic lymphangioma, pancreas, lymphangioma, distal pancreatectomy

## Abstract

Lymphangiomas are rare benign tumours of lymphatic vascular origin. They are more common in the paediatric population and manifest mainly in the neck and axillary region. Retroperitoneal lymphangiomas are <1% and pancreatic origin is even rarer. We present a case of a pancreatic cystic lymphangioma in a 60-year-old woman with chronic diffuse symptoms, diagnosed because of newly onset of diabetes mellitus. She was successfully managed with distal pancreatectomy and spleenectomy en-bloc with the cystic mass without any complications. Cystic lymphangioma of the pancreas is a rare entity presenting with a challenging preoperative diagnosis as imaging modalities may provide ambiguous information. The clinician should be aware of its complicated differential diagnosis and its persistent and subtle symptomatology.

## Introduction

Lymphangiomas are rare benign tumours of lymphatic vascular origin [1]. They occur due to a local obstruction of the lymphatic flow causing lymphangiectasia [2]. They are mostly congenital, in case of lymphatic obstruction during the foetal development, but they may be caused by trauma, fibrosis, inflammation and radiotherapy as well [3].

Lymphangiomas are more typical in the paediatric population and manifest mainly in the neck and axillary region in 75% and 20%, respectively. Retroperitoneal lymphangiomas are <1% and a pancreatic localization is even rarer [4].

Pancreatic lymphangiomas generally are incidentally discovered or present with subtle symptomatology [3]. Considering their rarity and non-specific symptoms or radiologic findings, preoperative diagnosis is a challenge. A biopsy may assist in the differential diagnosis from other cystic tumours of the pancreas, or in case of persistent or deteriorating symptomatology a straightforward surgical excision may take place [3].

We present a case of a pancreatic cystic lymphangioma in a 60-year-old woman diagnosed because of newly onset diabetes mellitus, with emphasis in the occult symptomatology and the therapeutic challenges. This case report was written in accordance with the SCARE criteria [5].

## Case presentation

A 60-year-old woman presented to the outpatient clinic with newly onset diabetes mellitus complaining of abdominal discomfort and mild epigastric pain for the past year. She had previously visited her endocrinologist who requested an upper abdomen magnetic resonance imaging (MRI). Her past medical history was remarkable for hypertension and tachycardia under medication and her newly acquired diabetes. Her past surgical history included an open appendectomy 52 years ago and a caesarean section 32 years ago. The patient was a moderate smoker and reported no allergies or alcohol intake. Her vital signs were normal while physical examination revealed a palpable upper abdominal mass along with mild epigastric tenderness upon palpation. Blood tests including white blood cells, serum amylase, and lipase as well as carcinoembryonic antigen (CEA) and cancer antigen 19-9 (Ca19-9) were within normal limits. The patient underwent an ultrasound (US) and a MRI before visiting our department and a computed tomography (CT) upon admission.

**Figure 1:**
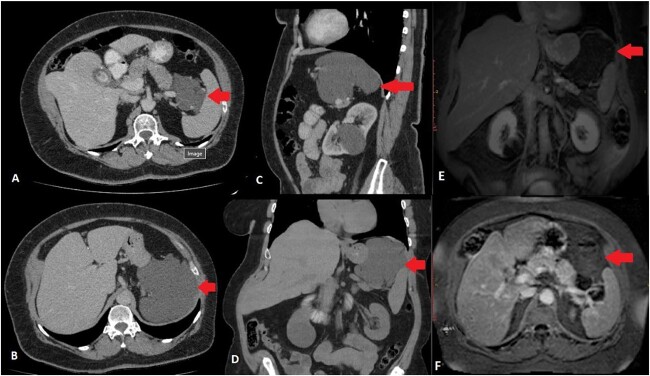
CT scan of the abdomen. Transverse scan—mass between pancreas and spleen (A), Transverse scan—mass expanding below the diaphragm (B), Sagittal scan—mass below the diaphragm and over the tail of the pancreas and splenic vessels (C), Coronal scan—mass between the diaphragm, upper pole of the spleen and tail of the pancreas (D), MRI scan of the abdomen. Coronal scan—mass between the diaphragm, upper pole of the spleen and tail of the pancreas, not enhancing (E), Transverse scan—mass between pylorus, pancreas, and spleen (F) ( arrow marking the mass).

Upper abdominal US revealed cholelithiasis and a large well-defined cystic lesion in close relation with the upper pole of the spleen and the tail of the pancreas measuring 11.6 cm in diameter. Abdominal CT showed a hypodense, cystic lesion with minimal enhancement between the tail of the pancreas and the spleen (Fig. 1). The mass was well-defined, measured 12 cm anterioposterior (AP) × 9.5 cm transverse (TR) × 10 cm cephalocaudal (CC), without any signs of infiltration of adjacent structures. There were no calcifications or soft tissue component. Abdominal MRI confirmed microcholelithiasis without signs of cholecystitis and intra- and extrahepatic bile ducts within normal range. It revealed multiple microcystic formations in the body and tail of the pancreas with possible communication with the main pancreatic duct giving the impression of branch-duct intraductal papillary mucinous neoplasm. Furthermore, between the tail of the pancreas and the splenic hilum, expanding to the left hemi diaphragm, a large multilobulated cystic lesion was described. The size of the lesion was 12.8 cm (AP) × 10.2 cm (TR) × 8.65 (CC), originating from the tail of the pancreas and displacing the spleen laterally and caudally and the stomach anteriorly (Fig. 1). The cyst did not present any solid component and no pathological signal intensity. There were no evident pathological lymph nodes or vascular invasion from the mass. MRI findings were suggestive for atypical pseudocyst, mucinous cystic neoplasm, or cystic lymphangioma.

**Figure 2:**
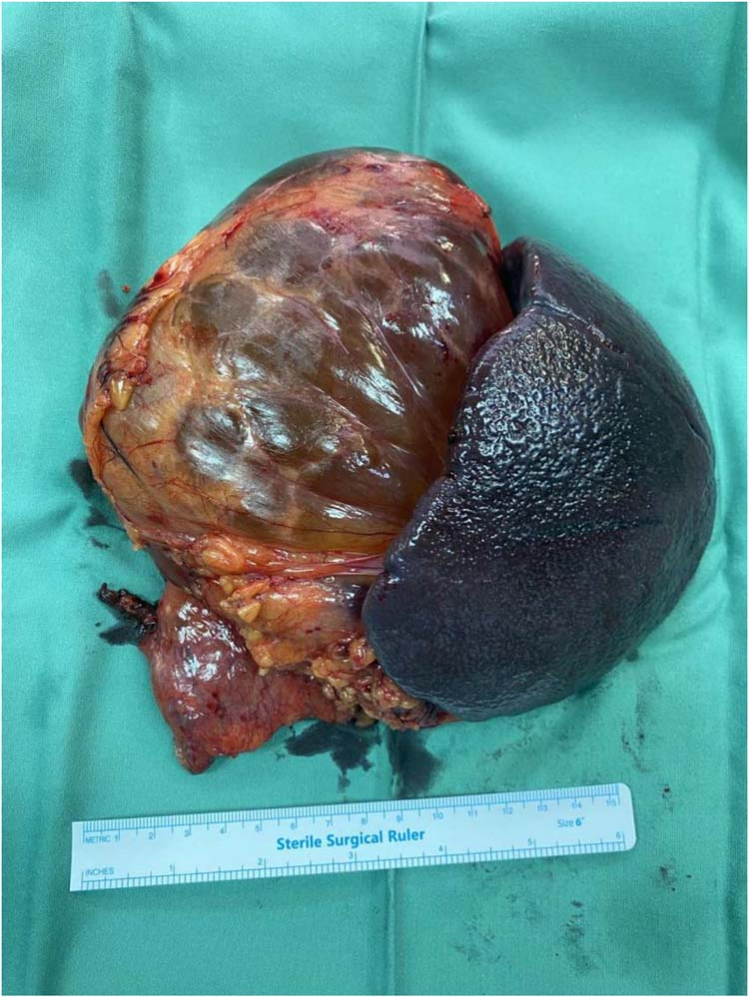
Surgical specimen of the distal pancreatospleenectomy en-bloc with the lymphangioma.

**Figure 3:**
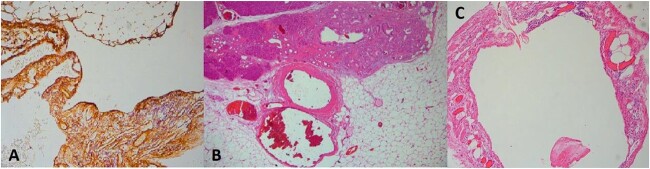
CD34 stain, ×100 (A), H–E, ×40 (B), and H–E, ×100 (C).

Due to the increased size and the long-lasting pressure symptoms of the cystic lesion, surgical exploration was decided at the multi-disciplinary team (MDT) meeting with the patient’s approval. The patient underwent exploratory laparotomy through a midline incision. The cystic mass was found to originate from the tail of the pancreas, adherent to the upper pole of the spleen. The mass was carefully mobilized from the adjacent structures and the resection was completed by en-bloc distal pancreatectomy with splenectomy and cholecystectomy (Fig. 2). The patient had an uncomplicated postoperative course and was discharged on post-operative day 5. Gross examination revealed a 13 × 11 × 7 cm well-described cystic tumour filled with yellowish fluid. On histopathological evaluation, the lesion consisted of anastomosing lymphatic spaces lined by a single layer of endothelial cells. The endothelial cells were positive for CD34, CD31, and D2-40 and negative for pankeratin (Fig. 3). The diagnosis of lymphangioma was made. There was no evidence of malignancy. Splenic and pancreatic tissues were found to be normal. Gallbladder specimen presented signs of chronic lymphocytic inflammation indicative for chronic calculus cholecystitis. MDT decision was follow-up. The patient is asymptomatic with no evidence of recurrence in 6 months.

## Discussion

Lymphangiomas are rare benign lymphatic tumours caused by congenital malformation of the lymphatic vasculature during the gestational period or by a lymphatic obstruction because of trauma, fibrosis, inflammation, or radiation [3]. They are more common among paediatric patients but due to the lack of symptomatology they are usually diagnosed in adult population. Common presentation sites include the neck and the axillary region in 95% of the cases and only 5% occurs in the mesentery, retroperitoneum, abdominal viscera, lung, or mediastinum [6]. Pancreatic lymphangiomas illustrate < 1% of all abdominal lymphangiomas and solely 0.2% of all pancreatic neoplasms.

Initially described by Koch in 1913 [7], pancreatic lymphangiomas are extremely uncommon with less than 100 cases reported in the literature [3]. A slight female predominance is reported with non-specific age distribution [8]. The size of pancreatic cystic lymphangiomas varies greatly in the literature, from 3 to 20 cm and an average size of 12 cm^2^. They may develop adjacent to the pancreas, within the pancreatic parenchyma, or connected to the pancreas via a pedicle [6]. A slight predisposition for the tail of the pancreas is also reported [8].

Patients with pancreatic cystic lymphangiomas are mainly asymptomatic. Small lymphangiomas are discovered incidentally. Larger lymphangiomas cause non-specific symptomatology due to their pressure effects which include abdominal pain and palpable mass upon examination. These symptoms were present in our case as well. Pancreatic lymphangiomas, especially large, may also cause acute abdominal symptoms if rupture, haemorrhage, or inflammation takes place which, however, is extremely rare [3]. There are no specific laboratory examinations for pancreatic cystic lymphangiomas and tumour markers are within normal limits in most of the cases [2].

Considering that symptoms, signs and laboratory tests are non-specific, imaging modalities portray an important role in the preoperative diagnosis. On US, cystic lymphangiomas present as multiple hypoechoic or anechoic cysts including multiple septa and rarely contain any echogenic material [8]. In our case, US revealed a single well-defined mass complicating the differential diagnosis. On CT, pancreatic cystic lympangiomas appear as well-defined, homogenous cystic masses with multiple septation which may show enhancement [6]. Microcalcifications because of phleboliths, haemorrhaging content with high attenuation or chyle are uncommon but may be present [4, 8]. MRI imaging generally reveal and strengthen US and CT findings. A cystic mass is observed with multiple septa which are enhanced after gadolinium administration. The cyst is hyperintense in T2 sequence and hypointense in T1 sequence [4, 6, 8]. Haemorrhage, infection or the presence of chyle may alter the cyst appearance on MRI, adding a solid component in its contents [4, 6, 8]. However, imaging studies are still non-specific for pancreatic cystic lympangiomas, as pancreatic mucinous and cystic lesions demonstrate similar features.

Therefore, accurate pre-operative diagnosis of a pancreatic cystic lymphangioma remains a challenge and multiple imaging modalities should be combined. Differential diagnosis would mostly include pseudocysts, serous, or mucinous cystadenomas, other congenital cysts and cystic ductal carcinoma [9].

In our case, the patient presented with non-specific symptoms of abdominal pain and a palpable mass. She also presented with new onset of diabetes mellitus which may not be related to pancreatic lymphangioma as it is with pancreatic cancer [10] but eventually led to the diagnosis of the cystic mass of the pancreas. Physical examination and laboratory tests were non-diagnostic and imaging modalities were inconclusive as the cystic characteristics were non-specific and even misguiding. Endoscopic US with fine-needle aspiration (EUS-FNA) was not performed because of the size of the lesion and patient’s symptoms as well as the possibility of malignancy or mucinous cystadenoma.

Treatment options for pancreatic cystic lymphangiomas, with established diagnosis, depend on their size and symptoms, as it does with other rare tumours of the pancreas like dermoid or epidermoid tumours [11]. If the patient is asymptomatic and the lesion does not cause pressure effects on adjacent organs, imaging surveillance may be embraced. If, as in our case, there is symptomatology due to the size of the cystic lymphangioma or there is a diagnostic dilemma concerning the nature of the lesion, surgical intervention is preferable. Surgical excision includes either simple cystectomy if feasible or more radical resections depending on the size of the lesion, its anatomic location within the pancreas and its relations with the adjacent organs [1]. In our case, the size of the pancreatic lymphangioma its location between the tail of the pancreas and the spleen as well as the diagnostic dilemma, contributed to the decision for distal pancreatectomy with splenectomy.

Upon pathology examination pancreatic cystic lymphangiomas are multilobulated and on cut section the smaller cysts interconnect representing multiple dilated lymphatic channels. Histologically, there are three types of lymphangiomas—cystic, capillary, and cavernous—but only the cystic and cavernous types have been reported in the pancreas. [8] Histologically, dilated lymphatic spaces along with attenuated endothelial cells are observed. [4] Immunohistochemically, lymphangiomas are positive for VIII-R antigen, CD31, and D2-40 and negative for CD34 [8] which however may occasionally be positive [12] as it presented in our case. They are negative for epithelial markers or periodic acid–Schiff stain [8].

In conclusion, cystic lymphangioma of the pancreas is a rare entity with limited data in the current literature. Pre-operative diagnosis is challenging as imaging modalities may provide ambiguous information. The clinician should be aware of the complicated differential diagnosis and the persistent subtle symptomatology, which usually leads to the operative theatre where surgical resection takes place. In the presence of preoperative suspicion and diagnosis along with a small sized and asymptomatic lesion, periodic imaging surveillance may be followed.

## Conflict of interest statement

The authors declare that there is no conflict of interest.

## Funding

This research received no funding.
